# Insights of antiparasitic activity of sodium diethyldithiocarbamate against different strains of *Trypanosoma cruzi*

**DOI:** 10.1038/s41598-021-90719-0

**Published:** 2021-05-27

**Authors:** Johny Wysllas de Freitas Oliveira, Taffarel Melo Torres, Cláudia Jassica Gonçalves Moreno, Bruno Amorim-Carmo, Igor Zumba Damasceno, Ana Katarina Menezes Cruz Soares, Jefferson da Silva Barbosa, Hugo Alexandre Oliveira Rocha, Marcelo Sousa Silva

**Affiliations:** 1 Immunoparasitology Laboratory, Department of Clinical and Toxicological Analysis, Centre of Health Sciences, Federal University of Rio Grande do Norte, Natal, Brazil; 2 Programa de Pós-Graduação em Bioquímica, Centro de Biociências, Universidade Federal do Rio Grande do Norte, Natal, Brazil; 3 Centro de Ciências Biológicas e da Saúde, Universidade Federal Rural de Semi-Árido, Mossoró, Brazil; 4 Programa de Pós-Graduação em Ciências Farmacêuticas, Centro de Ciências da Saúde, Universidade Federal do Rio Grande do Norte, Natal, Brazil; 5 Departamento de Engenharia de Materiais, Centro de Tecnologia, Universidade Federal do Rio Grande do Norte, Natal, Brazil; 6 Laboratório de Biotecnologia de Polímeros Naturais-BIOPOL, Departamento de Bioquímica, Centro de Biociências, Universidade Federal do Rio Grande do Norte, Natal, Brazil; 7 Instituto Federal de Educação, Ciência e Tecnologia do Rio Grande do Norte (IFRN), Campus São Gonçalo do Amarante, São Gonçalo do Amarante, Brazil; 8 Global Health and Tropical Medicine, Instituto de Higiene e Medicina Tropical, Universidade Nova de Lisboa, Lisbon, Portugal

**Keywords:** Drug screening, Target identification, Parasite biology

## Abstract

Chagas disease is caused by *Trypanosoma cruzi* and affects thousands of people. Drugs currently used in therapy are toxic and have therapeutic limitations. In addition, the genetic diversity of *T. cruzi* represents an important variable and challenge in treatment. Sodium diethyldithiocarbamate (DETC) is a compound with pharmacological versatility acting as metal chelators and ROS generation. Thus, the objective was to characterize the antiparasitic action of DETC against different strains and forms of *T. cruzi* and their mechanism. The different strains of *T. cruzi* were grown in LIT medium. To evaluate the antiparasitic activity of DETC, epimastigote and trypomastigote forms of *T. cruzi* were used by resazurin reduction methods and by counting. Different response patterns were obtained between the strains and an IC_50_ of DETC ranging from 9.44 ± 3,181 to 60.49 ± 7.62 µM. Cell cytotoxicity against 3T3 and RAW cell lines and evaluated by MTT, demonstrated that DETC in high concentration (2222.00 µM) presents low toxicity. Yet, DETC causes mitochondrial damage in *T. cruzi*, as well as disruption in parasite membrane. DETC has antiparasitic activity against different genotypes and forms of *T. cruzi*, therefore, representing a promising molecule as a drug for the treatment of Chagas disease.

## Introduction

Chagas Disease (CD) is a Neglected Tropical Disease (NTD) caused by flagellated protozoan *Trypanosoma cruzi*. This disease affects more than 8 million people worldwide, causing more than 10,000 deaths per year and has more than 80 million people living in risk zone^1^. CD presents an acute asymptomatic phase or nonspecific clinical signs, and a chronic phase, individuals may be asymptomatic or progress to cardiac and/ or digestive complications^2^. CD can be transmitted in different ways and the two main transmission mechanisms are vector transmission, during blood meal of triatomines, and ingestion of food contaminated with faeces of these vectors^3^. However, other transmission mechanisms also contribute to CD, such as blood transfusion, organ transplants, and vertical congenital transmission responsible for the dissemination of CD in other non-endemic countries^4^.

*T. cruzi* is a flagellated protozoan that belongs to the trypanosomatid family. The parasite has a complex heteroxenic life cycle that maintains its biological cycle between the invertebrate host of the *Triatominae* family and the mammalian host (humans, dogs, and wild animals) passing by metacyclogenesis during this cycle, suffering alteration in different forms^5^. Although *T. cruzi* is represented by a single species, the diversity and genetic variability of this parasite is made of different strains, grouped based on their genetic characteristics, named Discrete Typing Units (DTUs)^6^. Thus, *T. cruzi* can be classified into seven different genetic variants or genotypes, in which several strains were included, called from TcI to TcVI, and, more recently, the variant called TcBat^6,7^. These genetic variants of *T. cruzi* are important from an epidemiological point of view, as they are involved in different aspects of CD, mainly in the context of the transmission cycle, clinical manifestations, pathogenesis, reservoir and geographical distribution^8^.

In the context of pharmacological therapy for CD, the drugs currently used are benznidazole and nifurtimox^9^. These two nitroaromatic-compounds act on the parasite by producing reactive oxygen species (ROS), thus presenting antiparasitic activity. However, based on clinical point of view, these drugs have limitations regarding their use, since they are responsible for several adverse reactions like liver damage, pruritus, spots on the skin, and itchy in eyes^10^. Additionally, these drugs have low pharmacological efficacy in the chronic phase of infection with *T. cruzi*^11^. This fact has led to the search for new compounds that may be more effective in the treatment of CD.

Diethyldithiocarbamate (DETC) is a compound that belongs to the class of dithiocarbamates, formed by two ethyl substituents linked to an amine group, which in turn is linked to carbon disulfide. The chemical structure of DETC has two distinct portions; the first, an amine portion formed by nitrogen and the two ethyl groups that stimulate the production of reactive oxygen species. The second portion, a carbon disulfide end that can chelate on metals, thus attributing an essential biological activity of this compound against metalloproteases, enzymes necessary for parasitic biology^12–14^. Due to the structural characteristics of DETC, several pharmacological applications were observed in this compound^15–21^. In addition, we recently published a review that addresses the different justifications for enhancing DETC as a potential antiparasitic drug^15^.

However, little is known about the antiparasitic activity of DETC against *T. cruzi*. Thus, the aim of this study is to evaluate the antiparasitic activity of DETC in vitro against the different strains (different DTUs) and forms of *T. cruzi*. Therefore, in this work we report, for the first time, the antiparasitic activity of DETC against different strains of *T. cruzi*, as well as insights into its mechanisms of action as an antiparasitic drug.

## Results

### DETC in vitro antiparasitic activity towards *T. cruzi*

**Figure 1:**
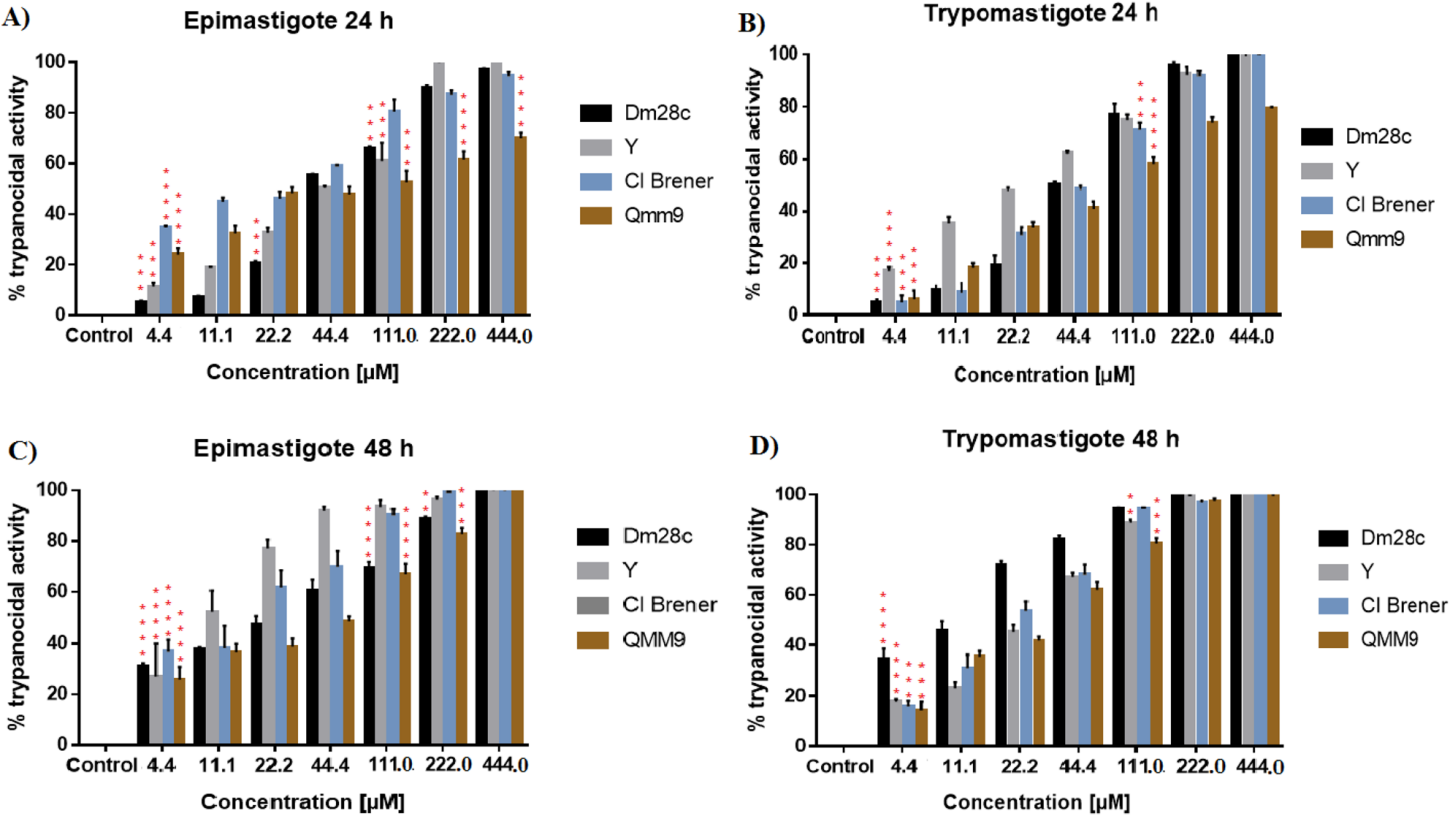
Evaluation of resazurin reduction inhibition by different strains and forms of *Trypanosoma cruzi* treated with DETC. Parasitic inhibition of resazurin reduction (%) of DETC against different forms (epimastigotes and trypomastigotes) and strains (Y, Dm28c, CL Brener, and QMM9) of *T. cruzi*, after 24 and 48 h of culture and analysed by resazurin assay. **(A)** Epimastigotes for 24 h of treatment; **(B)** Trypomastigotes for 24 h of treatment; **(C)** Epimastigotes for 48 h of treatment; **(D)** Trypomastigotes for 48 h of treatment. Results presented as mean ± standard deviation of the percentage of parasitic inhibition in a triplicate system and for the statistical analysis of the Anova Test, together with the Tukey Post-test (P < 0.01 (**); P < 0.001 (***), P < 0.0001(****). In order to verify differences, the profile of each strain was compared against others treated with the same concentration of DETC. Software GraphPad Prism v. 7.0 (2016) (https://www.graphpad.com/*)*.

The count of viable parasites by optical microscopy after treatment with DETC in different concentrations is presented in Fig. 1. Based on these results, it is possible to analyse that DETC causes a elevate toxicity against parasite depending of time of exposure and concentration applied. In addition, the effect of DETC is variable according with parasite form, in which epimastigote forms were most susceptible than trypomastigote forms.

QMM9 and CL Brener strains of *T. cruzi* demonstrated to be more susceptible than Dm28c and Y strains in low concentrations of DETC in the epimastigote form after 24 h of exposure. However, the increase of DETC concentration ended up affecting almost equally Dm28c, Y and Cl Brener strains, though the QMM9 strain was less affected in high concentrations of DETC. When epimastigote forms of *T. cruzi* were exposed 48 h at DETC, it was observed an accumulation of damage suffered by parasite. Even so, the QMM9 strain have been showed more resistance against DETC, but in higher concentration all strains do not present viable parasites.

The trypomastigote forms of parasite present a similar profile at epimastigote forms, but the Dm28c strain demonstrated a higher susceptibility at DETC than other strains. Furthermore, the trypomastigote forms present a higher resistance against DETC when compared with results achieved in epimastigote forms of *T. cruzi*. To confirm the antiparasitic activity of DETC against *T. cruzi*, the viability of parasites treated with DETC was also determined by resazurin metabolization. These results are shown in Fig. 1S. When comparing results presented in Fig. 1 with data in Fig. 1S, the same profile of DETC antiparasitic activity against *T. cruzi* is observed in both assays.

The IC_50_ of DETC was determined based on counting of viable parasites and values are shown in Table 1 and the results based on resazurin reduction are shown in Table 1S. The IC_50_ of DETC towards each strain of *T. cruzi* was different in the conditions tested, and the trypomastigote profile presented a higher IC_50,_ when compared with epimastigote, except for QMM9 strain. When compared with IC_50_ of benznidazole and tested in the same conditions of DETC, the IC_50_ of benznidazole was always higher for all strains in both forms.

**Table 1:** DETC antiparasitic activity, expressed in IC_50_ values ± standard deviation, against the different strains and forms of *Trypanosoma cruzi* after 24 h of exposure, based on results expressed by couting essay.

*Trypanosoma cruzi* strains (DTUs)	IC_50_ epimastigote		IC_50_ trypomastigote	
	DETC (μM)	BZN (μM)	DETC (μM)	BZN (μM)
Strain Dm28c (TcI)	37.13 ± 7.58^a^	78.12 ± 8.01^a^	40.12 ± 6.58^a^	91.95 ± 2.87^b^
Strain Y (TcII)	50.41 ± 3.56^b^	96.66 ± 5.38^b^	31.19 ± 7.21^b^	98.74 ± 3.98^b^
Strain QMM9 (TcIII)	60.15 ± 6.45^b^	101.38 ± 5.39^b^	46.76 ± 5.11^a^	103.47 ± 2.45^c^
Strain CL Brener (TcVI)	49.12 ± 5.78^b^	71.56 ± 7.98^a^	43.83 ± 4.55^a^	82.43 ± 5.83^a^

The statistical analysis the Anova Test together with the Tukey Post-test. The statistical analysis used measure the IC_50_ value in each group under same conditions.

*BZN* benznidazole.

The QMM9 strain was less affected by exposure to DETC, it showed the highest IC_50_ among the tested strains. Meanwhile, with the smallest IC_50_, the Dm28c strain was the most affected by exposure to DETC. The Y and CL Brener strains have similar profiles in epimastigotes, and different responses in trypomastigotes. Furthermore, the Y, QMM9 and CL Brener strains have a lower IC_50_ in trypomastigote form when compared with the result in epimastigote form. At the same time, QMM9 strain had a reduced in IC_50_ of epimastigote form compared with the trypomastigote form.

In addition, the correlation between each IC_50_ result of DETC and benznidazole against all strains in different forms was also determined. Based on the results obtained, a R square > 0.88 to all strains tested was observed through the Pearson Correlation, representing a strong correlation between results of DETC and benznidazole. The effectiveness of the anti-parasitic activity of DETC was also compared with the effectiveness of benznidazole for each strain and evolutionary form evaluated in this study.

Additionally, in order to validate the effectiveness of the DETC antiparasitic activity against *T. cruzi* strains, the parasites treated with DETC and with 100% resazurin reduction capacity were re-cultivated in a new culture medium, and after 7 days of culture these parasites had no cell viability (results not shown).

### Visualization of DETC damage towards *Trypanosoma cruzi*

**Figure 2:**
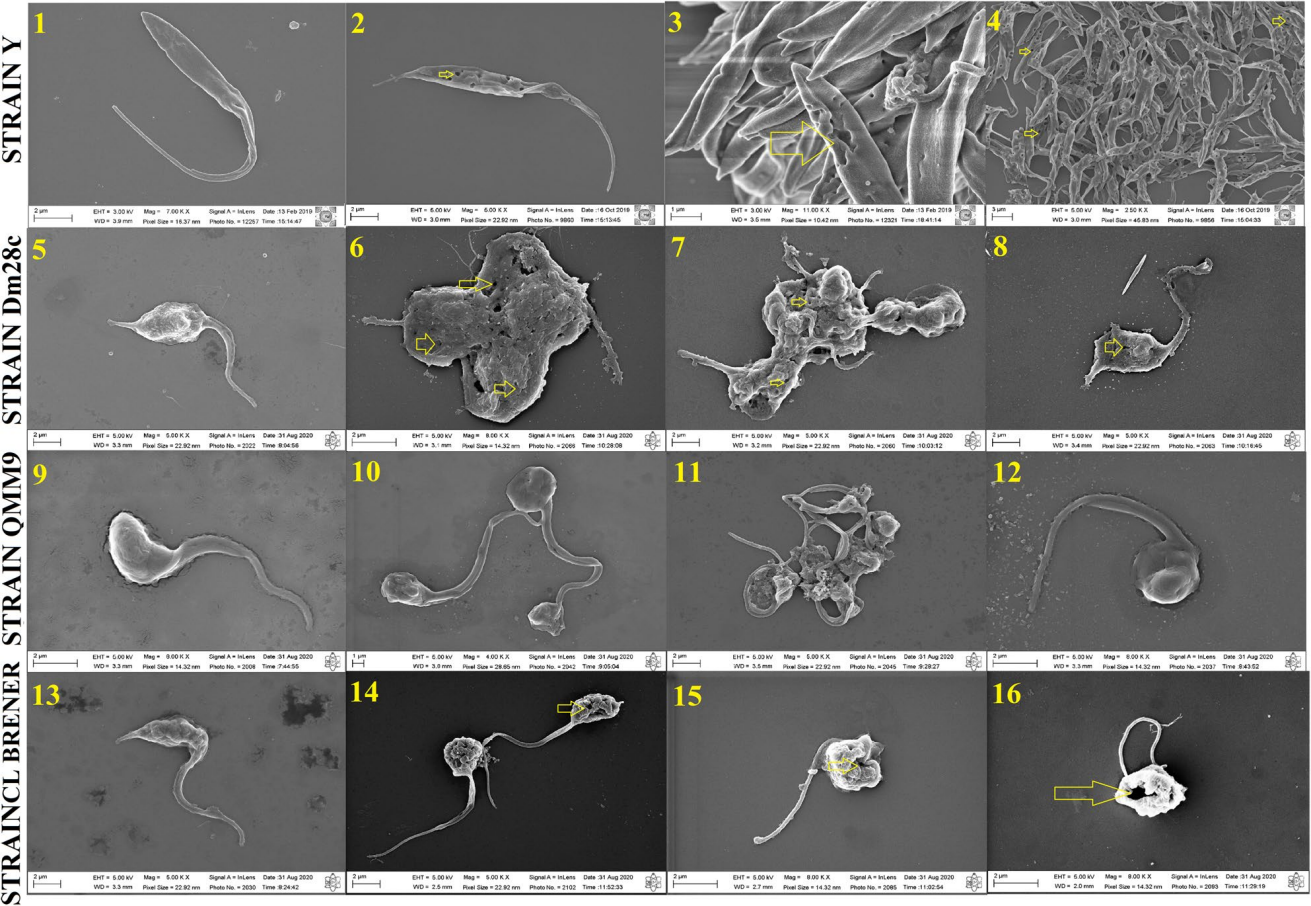
Morphological changes of *Trypanosoma cruzi* epimastigotes treated with diethyldithiocarbamate and analysed by scanning electron microscopy. Image 1 corresponding to the control of strain y, 2—4 corresponding to the treatment with DETC in concentrations 44 µM (2), 111 µM (3) and 222 µM (4); image 5 corresponding to the control of strain Dm28c, 6—8 corresponding to the treatment with DETC in concentrations 44 µM (6), 111 µM (7) and 222 µM (8); images 9 corresponding to the control of strain QMM9, 10—12 corresponding to the treatment with DETC in concentrations 44 µM (10), 111 µM (11) and 222 µM (12); and image 13 corresponding to the control of strain CL Brener corresponding to the treatment with DETC in concentrations 44 µM (14), 111 µM (15) and 222 µM (16), 14—16 . The yellow arrows in the figure indicate the presence of pore in parasite structure. Scanning electron microscopy under a FEG microscope (Model augira, Brand Carl Zeiss, Oberkochen, WB, GER).

In order to observe if the exposure to DETC by different strains of *T. cruzi* causes morphological changes in the parasite membrane structure after treatment, the parasites had their membrane structure analysed by scanning electron microscopy (SEM). Figure 2 has four columns; the first column shows the images 1, 5, 9, and 13 corresponding to the control of Y, Dm28c, QMM9 and CL Brener strains, respectively. These images show the normal morphology of the parasite without treatment with DETC. The remaining images present in the second, third and fourth columns show the parasites treated with DETC. Three images of each strain treated with DETC were used to visualize the damage suffered by *T. cruzi* treated with DETC. Based on the images it was possible to observe different alterations suffered by the parasite. In the Y strain of *T. cruzi* (images 2, 3, and 4) we observed pores in the membrane in large scale. In the Dm28c strain of *T. cruzi* (images 6, 7, and 8) we observed a large damage in the parasite membrane and the presence of pores, but they are fewer than in the Y strain of *T. cruzi*. In the QMM9 strain of *T. cruzi* (images 10, 11, and 12) we observed alterations in the membrane morphology, lacks in the membrane, disruptions and small pores. Finally, in the CL Brener strain of *T. cruzi* (images 14, 15, and 16) we observed big pores and alterations in the parasite membrane morphology.

EDS analysis was used as a quality control for the structures shown in the SEM images (Fig. 2S). The carbon structure of the parasites inserted in the correct place on the silicon plate and low salt concentration were observed in the samples used in the SEM analysis. For the images used in the SEM analysis, an EDS analysis was performed to verify and confirm the results obtained.

### DETC cytotoxicity in vitro cell culture

**Figure 3:**
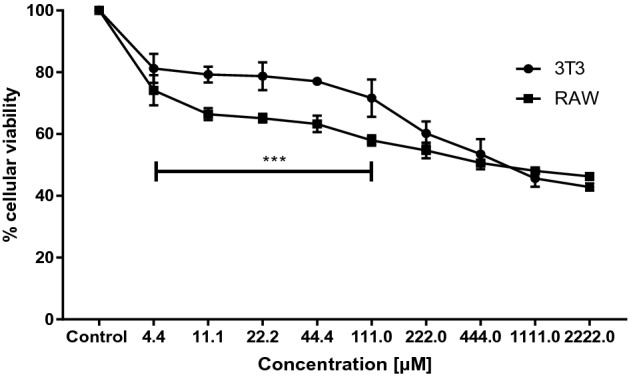
Evaluation of the MTT reduction in 3T3 and RAW cell lines exposed to diethyldithiocarbamate. Viability curves of the 3T3 and RAW cell lines obtained through the MTT reduction assay when treated with different concentrations of DETC for 24 h. 100% of MTT reduction represents cells untreated. The curves present in this image were constructed based on mean ± standard deviation of the percentage of MTT reduction for each cell line (RAW and 3T3). To compare the MTT metabolization of each cell lines against same concentration of DETC treatment, a statistical analysis was performed using T de student Test. The statistical analysis showed difference in metabolization on range concentrations from 11.1 to 44.4 µM P < 0.001 (***). Software GraphPad Prism v. 7.0 (2016) (https://www.graphpad.com/).

The next step was to assess the DETC effect on the ability of mammalian cells to reduce MTT. Therefore, RAW and 3T3 cell lines were submitted to DETC (from 4.44 to 2222.0 µM) for 24 h, as described in the methods section. As shown in Fig. 3, DETC affected the cells' ability to reduce MTT. Low concentrations of DETC affected RAW more than 3T3 cell lines. From the concentration of 11.1 µM to 111.0 µM, the RAW cells was more sensitive, statically observed from concentration 222.0 µM, RAW and 3T3 cell lines had similar profiles of response against DETC. In this case, the ability of both cells to reduce MTT decreased to around 50%, and did not decrease, even with an increase in the concentration (from 1111.0 to 2222.00 µM) of DETC. The IC_50_ of each cell line was determinated based on the decrease of reduce MTT when exposed to DETC. Then, based on the MTT metabolization curve, present in Fig. 3, for each cell line, the IC_50_ values were calculated, 859.90 µM and 698.80 µM for cellular lineages, 3T3 and RAW, respectively.

The selectivity index (SI) was determined based on the data from the resazurin and the MTT assays, as described in the Methods section. Table 2 shows the different SI of DETC obtained with different strains in trypomastigote form of *T. cruzi*. In addition, the different strains associated with each cell line presented different SI. The selectivity of DETC for the QMM9 strain towards the cell lines 3T3 and RAW, presented the smaller values of SI for both forms and cellular lineages. Meanwhile, selectivity for the Y strain towards cell lines presented higher values of SI and cellular lineages. Furthermore, the SI of DETC for the CL Brener and QMM9 strains presented a similar profile.

**Table 2:** Selectivity index (SI) of diethyldithiocarbamate in different strains in trypomastigote form of *Trypanosoma cruzi* after 24 h of cultivation

*Trypanosoma cruzi* strains (DTUs)	3T3: Trypo	RAW: Trypo
Strain Dm28c (TcI)	34.396	27.952
Strain Y (TcII)	36.827	29.927
Strain Qmm9 (TcIII)	16.076	13.064
Strain CL Brener (TcVI)	19.914	16.183

Different forms of *T. cruzi* epimastigote (Epi) and trypomastigotes (Trypo) and cell lines (3T3 and RAW cells).

### Mechanisms of cellular death in *Trypanosoma cruzi* after treatment with DETC

**Figure 4:**
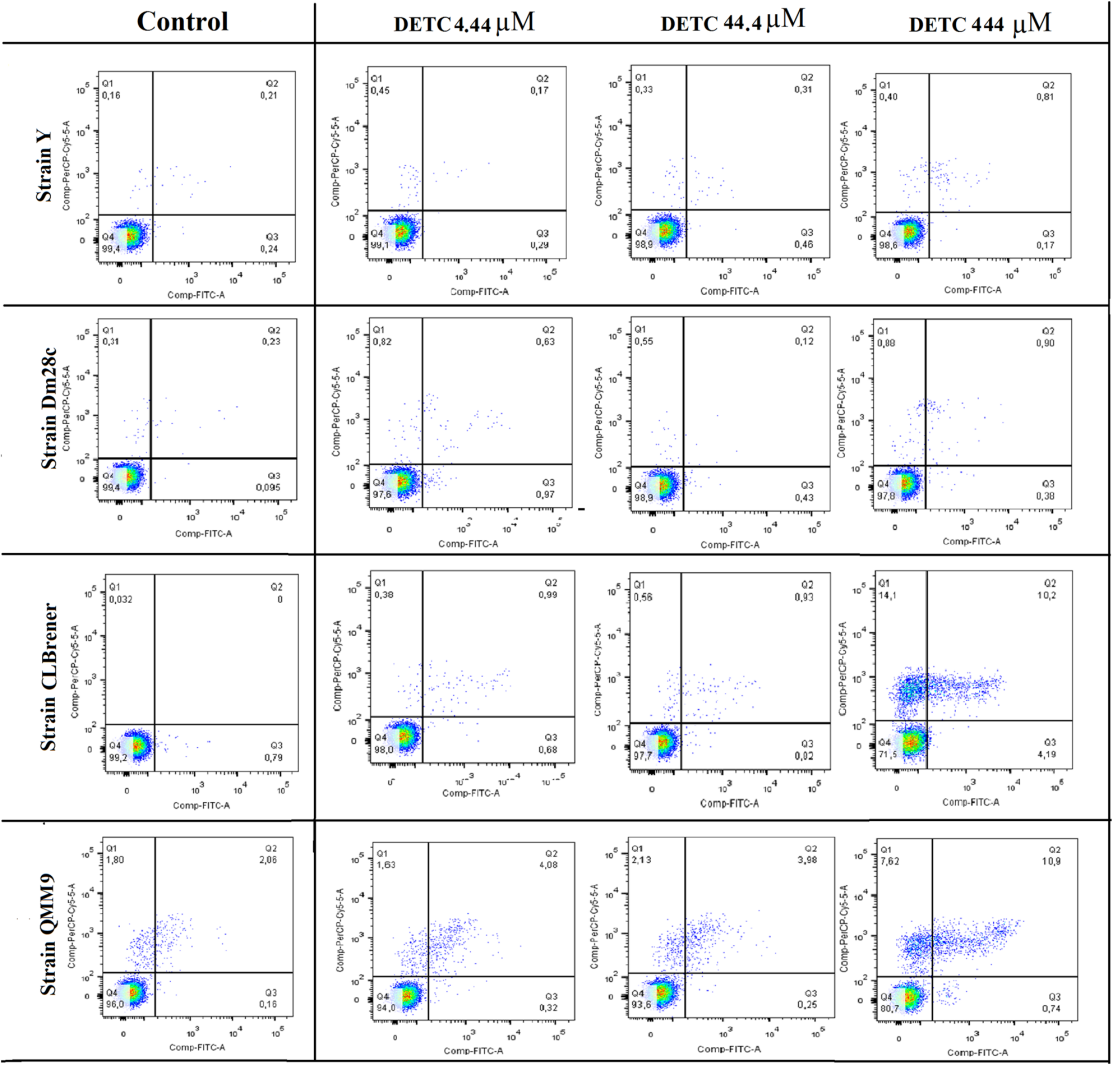
Analysis of the cell death mechanism of trypomastigotes from different *Trypanosoma cruzi* strains treated with DETC. The parasites death cell mechanism was evaluated by flow cytometry as to the capacity for Annexin/PI stain after 24 h of treatment with different DETC concentrations; 4.44 µM; 44.44 µM; and 444.00 µM. Flow cytometer (FACSCanto II, BD Biosciences, Eugene, OR, USA) with FACSDiva software, version 6.1.2 (Becton Dickson, Franklin Lakes, NJ, USA) (https://www.bdbiosciences.com/en-us/instruments/research-instruments/research-software/flow-cytometry-acquisition/facsdiva-software).

Annexin V/PI assay, markers of cellular death, was analyzed towards different forms and strains of *T. cruzi* when treated with DETC. The profile of trypomastigote forms treated with DETC is shown in Fig. 4 and the epimastigote form in Fig. 3S. Based on these results it is possible to observe the absence of death cellular markers Annexin V/PI in all strains of *T. cruzi*, when exposed to different concentrations of DETC (4.44 µM, 44.40 µM, 444.00 µM). These concentrations were used based on results of resazurin and counting of parasites, seeking to identify the mechanism of death related to the stain Annexin V/PI by flow cytometry.

Data for Y and Dm28c strains of *T. cruzi* when treated with DETC was similar to those observed with untreated parasites, that is, more 97% of parasites were considered viable, even when DETC was used at higher concentration (444.00 µM). However, a low fluorescence response of death cellular markers Annexin V/PI was verified to CL Brener and QMM9 strains of *T. cruzi*, when exposed to highest concentration of DETC. 71% and 80% of viability was identified for both strains after exposure, respectively.

### Mitochondrial damage in *Trypanosoma cruzi* after treatment with DETC

A second possible death cell mechanism was evaluated by the mitochondrial activity assay using rhodamine-123. In this case, the parasites were treated with different concentrations of DETC (44.4 µM; 111.0 µM; and 222.0 µM) and subsequently, the fluorescence rhodamine-123 stain was detected by flow cytometry. These concentrations were used to visualize the effect of DETC towards the parasite exposed for 24 h. The concentration of 444.0 µM DETC was not used to prevent the death of 100% of the parasites, and thus making the rhodamine-123 assay unfeasible.

**Figure 5:**
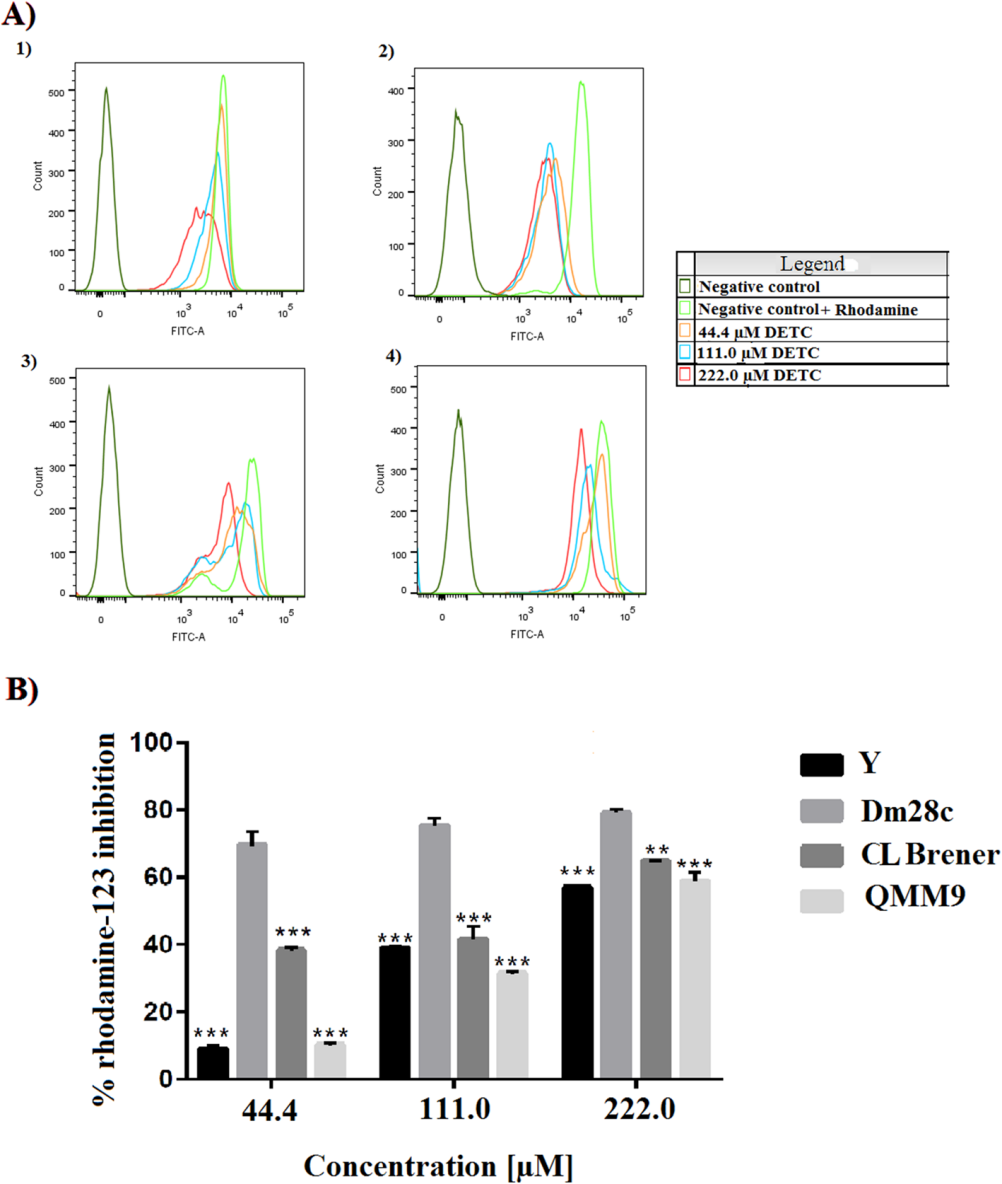
Evaluation of the mechanism of mitochondrial damage caused by DETC in *Trypanosoma cruzi*. **(A)** Fluorescence intensity (rhodamine-123) in different strains in trypomastigote form of *T. cruzi* untreated (control), represents 100% of fluorescence, and treated with different concentrations of DETC (44.4 µM, 111.0 µM, 222.0 µM) for 24 h. (1) Y strain; (2) Dm28c strain; (3) CL Brener strain; (4) Qmm9 strain. 10,000 events were used for each analysis to obtain the data, which was carried out in triplicate. **(B)** Fluorescence intensity (%) of rhodamine-123 inhibition of different strains of *T. cruzi* treated with different concentrations of DETC. The results were presented based on mean ± standard deviation of the percentage of parasitic inhibition. The statistical analyses were performed using the Anova Test with the Tukey Post-test (P < 0.01 (**); P < 0.001 (***). The statistical analyses were used to compare the profile of each strain treated with the same DETC concentration. Flow cytometer (FACSCanto II, BD Biosciences, Eugene, OR, USA) with FACSDiva software, version 6.1.2 (Becton Dickson, Franklin Lakes, NJ, USA) (https://www.bdbiosciences.com/en-us/instruments/research-instruments/research-software/flow-cytometry-acquisition/facsdiva-software) and software GraphPad Prism v. 7.0 (2016) (https://www.graphpad.com/).

The fluorescent profile of rhodamine-123 in each *T. cruzi* strain when exposed to DETC is shown in Fig. 5A. The results shown in Fig. 5A were quantified and used to produce Fig. 5B. All *T. cruzi* strains treated with DETC, when compared to the control, show a reduction in the fluorescence profile for rhodamine-123. The increase in DETC concentration affects the fluorescence intensity of the parasite for rhodamine-123 by flow cytometry. Y and QMM9 strains of *T. cruzi* were the least susceptible to DETC, with a similar profile of reduction in fluorescence emission, and the Dm28c strain was the most susceptible to DETC, resulting in smaller fluorescence when compared with control.

### Reactive oxygen species production in *Trypanosoma cruzi* after treatment with DETC

**Figure 6:**
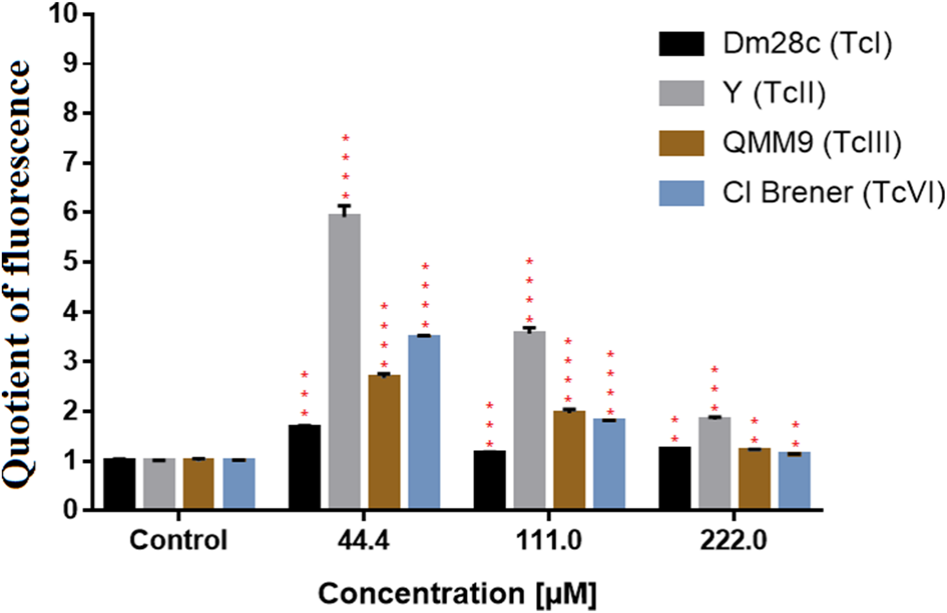
Evaluation of reactive oxygen species production in trypomastigote forms of *T. cruzi* after 24 h of exposure at DETC. The different strains of *T. cruzi* were treated with 4 concentrations of DETC (22.2 µM, 44.4 µM, 111.00 µM and 222.00 µM. Results presented as mean ± standard deviation of the percentage of parasitic inhibition in a triplicate system and for the statistical analysis the Anova Test together with the Tukey Post-test P < 0.0001(****) when compared with control. Software GraphPad Prism v. 7.0 (2016) (https://www.graphpad.com/).

The reactive oxygen species (ROS) production in trypomastigote forms of *T. cruzi* after treatment with DETC for 24 h are shown in Fig. 6. The results demonstrated, when compared with control all strains after treatment with DETC, an increase in reactive oxygen production. The Dm28c and Y strains were most affected after treatment with DETC when analysed the increase of ROS. However, the increase of concentration after 44.4 µM presented a reduction of ROS production when compared with previous concentration. The QMM9 and Cl Brener strains presented a similar profile of ROS production.

## Discussion

This work evaluated the antiparasitic activity of DETC against four different strains of *T. cruzi*: Dm28c (TcI), Y (TcII), QMM9 (TcIII), and Cl Brener (TcVI) strains. The antiparasitic activity data of DETC (Fig. 1, Fig. 1S and Table 2) show that different strains of *T. cruzi* have different levels of susceptibility. Previously, dithiocarbamate was classified as a group compounds with high activity against *T. cruzi*. Studies have shown the antiparasitic activity of different compounds based on dithiocarbamates against *T. cruzi*, probably due to the inhibition of the enzyme Superoxide Dismutase enzyme (SOD) and other molecules of the oxidative pathway of the parasite^22,23^. Furthermore, the different inhibitory resazurin profile and counting for different strains after treatment with DETC is in agreement with results obtained by others authors. They notice that different patterns of response should be associated with phylogenetic and evolutionary processes that act making the strains resistant or susceptible at compound^24–26^.

The experiments performed in this work, based on counting and resazurin reduction, used concentrations different of IC_50_, adopting as pattern 44.4 µM, 111.0 µM, and 222.0 µM, except the annexinV/PI have been used 4.44 µM, 44.4 µM and 444.00 µM to demonstrate that the concentration applied almost not change the result present, whether in high concentrations or lower concentrations. In the others experiments, the approach that we came here, is based in different profile observed in results between strains of *T. cruzi*. The use of different concentrations allowed observe and have a better interpretation of how the strains of parasite response under different concentrations of DETC and find better explanations.

In addition, a different profile of inhibition was demonstrated when the alteration in evolutionary form of *T. cruzi* occurs*,* in this case, the epimastigote and trypomastigote forms. The different profiles in evolutionary forms towards treatment with DETC are associated with metacyclogenesis processes. In these processes, there is an alteration in protein expression, RNA and changes in the parasite structure, which acts directly or indirectly in the strain resistance at compound^27–29^.

It is worth mentioning that QMM9 strain showed the more resistant profile in the DETC treatment and the same was observed when exposed to the conventional drug used in CD treatment (benznidazole)^30^. However, this strain was recently isolated and characterized, and therefore there are no studies with mechanisms or causes for this high resistance.

Our results show that DETC has a better antiparasitic activity against *T. cruzi* than benznidazole, when compared in vitro and in the experimental conditions established in this study. In another study, it was observed that benznidazole presents an IC_50_ < 10 μM against Y strain of *T. cruzi*^31^. However, here we used a higher concentration of parasites for antiparasitic assay (10^7^ parasites/mL). In the other study the antiparasitic activity was determined in a lower concentration of parasites (10^6^ parasites/mL). In addition, we analysed the antiparasitic activity of DETC and benznidazole in shorter exposure times (24 and 48 h), while many other authors use benznidazole treatment in 72 h^31,32^.

To evaluate the damage caused by DETC in parasite structure, SEM analysis was used after treatment with DETC for 24 h. It was observed that DETC causes severe damage in parasite structure and acts in pore formation (Fig. 2). In the literature, DETC was characterized as an inhibitor of antioxidative enzymes, causing the accumulation of reactive species inside cell or parasite. The excessive presence of reactive species causes a lot of damage and leads to the formation of pores. In others studies that used a compound able to trigger the same increase of reactive species, the formation of pores was in SEM analysis and membrane damage^33,34^.

With regard to the DETC cytotoxic profile, in this study we observed the viability of the 3T3 and RAW cell lines treated with DETC for 24 h (Fig. 3). Only in high concentrations (1111.0 μM and 2222.0 μM), DETC was able to significantly reduce the viability of the 3T3 and RAW cell lines, but not less than 40% of cell viability. Even at high concentrations, DETC was not toxic enough to cause 100% mortality of the cell lines. However, even in low concentrations, DETC has 100% antiparasitic activity. With these data it was also possible to determine the selectivity index (SI) of DETC of different strains and forms of *T. cruzi* (Table 2). Based on the SI, DETC showed less toxicity for fibroblast cell lines when compared to benznidazole and derivatives^35^. In addition, DETC has less toxicity than benznidazole when evaluated in RAW cell lines^36^. These results corroborated the evaluation of in vitro DETC efficacy compared with conventional drugs used in treatment of CD.

In this work, we take an approach showing DETC as a potent inhibitor of *T. cruzi* activity. In others studies using DETC against parasites of Trypanosomatidae family^20,21^, DETC showed an important Fe-SOD inhibitor resulting in O^-2^ accumulation and causing the parasite to die. In vitro studies have also shown that DETC can inhibit Fe-SOD of *T. cruzi*^22,23^. Therefore, when analyzing the stain of Annexin V/PI (Fig. 4 and Fig. 3S), the absence or low staining for both evolutionary form and for different strains of parasite was observed. This result was expected, has it was observed that SOD inhibitors can blocked apoptosis pathway and denied the cascade signalization to expose phosphatidylserine in membrane, since the stain was not detected^37,38^. Furthermore, studies with dithiocarbamates showed compound’s capacity to inhibit the apoptosis pathway and induce non-specific necrosis^39^.

The structure of DETC may act by producing reactive species and causing inhibition of antioxidative enzymes. Inside cells and parasites, the site most commonly associated with reactive species production and neutralization is the mitochondria. Therefore, in our work we evaluate whether DETC could attack and damaged the mitochondria. When observing the results present in Fig. 1S and Fig. 5, both resazurin and rhodamine-123 are metabolized inside the mitochondria. It is known that the metabolism of these two compounds by the parasite is directly related to the functioning of complexes I, II and III of the inner membrane of the mitochondria^40^. The reduction phenomenon of the resazurin and rhodamine-123 compounds may be related to the damage of the mitochondrial inner membrane of *T. cruzi* due to the action of DETC. Consequently, causing a malfunction in the regulation of the protonation gradient carried out by the mitochondrial complexes I, II and III, and impairing ATP synthesis, and subsequently causing the death of the parasites^40–43^. Interestingly, it can be seen in Fig. 5 that the highest concentration of DETC (222.0 µM) is able to reduce the metabolism of rhodamine-123 in all strains of *T. cruzi* analysed in this study, probably associated with the reduction of the membrane potential mitochondrial, potentiating the mitochondria as a possible target for the anti-*T.cruzi* activity presented by DETC.

*T. cruzi* treated with DETC shows damage and pore formation in the cytoplasmic membrane. This phenomenon can also be a consequence of mitochondrial damage induced by DETC. Some studies report the capacity of mitochondrial damage caused by the production of reactive species, a possible explanation for the antiparasitic mechanism presented by DETC^44–46^.

When analysing the results present in Fig. 6 demonstrating the increase in ROS in *T. cruzi* according to the exposure at DETC in different concentrations. The different profiles present by parasites are in agreement with the results presented in others experiments. Strain Dm28c proved to be more susceptible in the trypomastigote (Fig. 1) form and suffered higher mitochondrial damage in low concentrations of DETC (Fig. 5), these results may be associated with a higher ROS production in lowers concentrations of DETC, observed in Fig. 6. The reduction of ROS production by all strains after treatment, observed in highest concentrations (111.00 µM and 222.00 µM), is associated with high death of parasites after treatment with DETC in these concentrations (Fig. 1), due to the reduction of the ROS production level in these concentrations. Furthermore, the results presented in Fig. 6 corroborate with others results present in this work and reinforce the idea of the importance of ROS damage for the parasite elimination.

In addition, when observe the ROS production by parasite exposed at DETC in different concentrations, the increase of ROS present a similar profile than other classes of compounds tested against *T. cruzi* that present the same mechanism. Nevertheless, studies using benznidazole and derivatives present results with lower ROS production in 24 h when compare with results demonstrated in this work. Therefore, DETC present an interesting result in ROS production against different strains of *T. cruzi* corroborating with other authors those use compounds with capacity of stimulate ROS production^55–57^.

In conclusion, in this work we demonstrated the antiparasitic activity of DETC against different forms (epimastigotes and trypomastigotes) and four different strains of *T. cruzi*, the etiological agent of CD. When we compared the antiparasitic activity of DETC with benznidazole, the first-line drug for the treatment of CD, DETC showed better antiparasitic activity in vitro. Finally, new assays and validations are necessary in order to enhance the use of DETC as a promising candidate in the treatment of Chagas disease.

## Methods

### Axenic cultivation of *Trypanosoma cruzi*

Different strains of *T. cruzi* TcI (strain Dm28c)^47^; TcII (strain Y)^48^; TcIII (QMM9 strain)^30^ and TcVI (CL Blener strain)^49^ were grown in LIT (Liver Infusion Tryptose) medium supplemented with 10% inactivated fetal bovine serum (FBS) (v/v) and 5% of antibiotic streptomycin/penicillin (100 UI/mL). All strains were donated by *Laboratório de Parasitologia* (FCFAr)*,* Araraquara, Brazil. The epimastigote forms of *T. cruzi* were kept at 27 ºC in a BOD oven (incubator chamber, ASP, SP-500). The trypomastigote forms of *T. cruzi* were obtained through the nutritional stress method, from the epimastigote forms of the parasite for a period of 25 days, according to the methodology developed by Camargo et al., 1964^50^. Briefly, 20 mL of cell culture were kept in 50 mL flasks. The flasks were kept closed and the parasites were subsequently analysed for cell viability and differentiation morphological changes. The morphological changes of the parasites were analysed by optical microscopy, and a parasitic concentration greater than 75% of the trypomastigote forms was considered.

### Cells

RAW 264.7 macrophages (ATCC number TIB-71) and 3T3 fibroblast (ATCC CRL-1658) cell lines were cultured in DMEM (Cultilab, Campinas, SP, Brazil) supplemented with 10% (v/v) fetal serum bovine (FBS) (Cultilab, Campinas, SP, Brazil) and antibiotics (100 U/mL penicillin and 100 μg/mL streptomycin). The cells were incubated at 37 C in a humidified atmosphere with 5% CO_2_. For maintenance of the cells, the culture medium was changed every three days, and the cells were further subcultured at 80% confluence using a cell scraper (RAW cells) or trypsin/EDTA (3T3 cells).

### Determination of DETC antiparasitic activity against different strains and forms of *Trypanosoma cruzi*

The different strains and forms of *T. cruzi*, mentioned above, were used in this study. The parasites were diluted in a concentration of 1.0 × 10^7^ parasites/mL and then 200 µL kept in 96-well plates together with different concentrations of sodium diethyldithiocarbamate (DETC) solved in LIT medium, ranged from 4.44 to 444.00 µM. The antiparasitic activity of DETC was determined through the viability of the parasites after a treatment period of 24 and 48 h. Benznidazole was used as a positive control of antiparasitic activity against *T. cruzi*, in different concentrations that ranged from 3.84 to 500.00 µM. This range was selected due to an existing variability response against the different strains of *T. cruzi* and to determinate the IC_50_ of benznidazole to compare with DETC.

Parasite viability was determined by the resazurin (Sigma Aldrich, Laramie, WY, USA) reduction assay at a concentration of 1 mM and subsequently measured by spectrophotometer (Epoch, BioTek Instruments, Winooski, VT, USA), at 570 nm and 600 nm wavelengths^51^. The antiparasitic activity of DETC was expressed as % of resazurin reduction = 100 – [(Atest570 – (Atest600*Ro))/(Acontrol570—(Acontrol600*Ro)] × 100, in which Atest corresponds to the absorbance of the experimental group, and Acontrol corresponds to the absorbance of the negative control, 570 and 600 are the wavelengths that correspond to 570 nm and 600 nm, Ro represents the index of correction between the medium and resazurin, that allows to analyse if DETC interfere in the resazurin activity used during the test. It was observed that DETC does not interfere in the resazurin activity. Based on the percentage of reduction in resazurin, the IC_50_ of the compounds were calculated, representing the concentration of the drug needed to reduce the viability of a parasitic population by 50%.

The viability of the parasites was also determined by microscopy in a clear camera through direct counting in a Neubauer camera. In this assay, parasites with some movement were considered viable. Cell viability results were compared between the two methodologies. In some conditions, when the DETC concentration produced 100% of parasite mortality, they were removed from the culture medium and later centrifuged (2,000 rpm, 10 min, 4 C) to remove the drug. Afterwards, the parasites received a new-LIT medium, supplemented with 10% (v/v) FBS and 5% of antibiotic streptomycin/penicillin (100 UI/mL). After 7 days, the culture was analysed by conventional optical microscopy. The absence of live parasites confirms 100% mortality.

### Evaluation of the membrane structure alteration in *Trypanosoma cruzi* submitted to DETC by scanning electron microscopy

To evaluate possible morphological changes in the membrane of different strains of *T. cruzi* caused by DETC, the parasite membrane structure was analysed by scanning electron microscopy after treatment with DETC, according to a protocol established by Amorim-Carmo et al., 2019^52^. Briefly, the epimastigote forms of the different strains of *T. cruzi* (1.0 × 10^7^ parasites/mL), in the same cultivation conditions described previously in topic 2.2, were treated with DETC (44.40 µM, 111.00 µM and 222.00 µM), also in identical conditions described previously. After 24 h, the parasites were centrifuged for 10 min at 2000 rpm (4 C) and washed twice in a saline solution (0.9% NaCl) pH 7.4. The parasites were then fixed in solution of 2.5% glutaraldehyde in PBS (v/v) for 4 h. After this step, the parasites were dehydrated, being exposed to different ethanol concentrations. First, to dehydrate the parasite, ethanol at 25% was added and left to stand for 10 min, followed by washes in a saline solution (0.9% NaCl) pH 7.4 for 10 min at 1500 rpm room temperature. The same operation was repeated for 50%, 80%, and 100% ethanol concentrations to dehydrate the parasite for next steps. Then, the parasites were resuspended in absolute ethanol, discarded on silicon plates and placed to dry at room temperature. Finally, once dry, the parasites were placed on stubs and subjected to metallization with gold using the sputtering (Bal-Tec SCD-005 Sputter Coater, Schalksmühle, NWR, GER) in an argon atmosphere for 30 s with a current of 30 mA, and then analysed by a scanning electron microscopy under a FEG microscope (Model augira, Brand Carl Zeiss, Oberkochen, WB, GER). In addition, an X-ray spectroscopy for energy dispersion (SED) was performed, in order to validate the results in the SEM. This technique allows the visualisation of the elements present in the samples, the structure and components of parasites, and allows identification of the presence or absence of residues and impurities.

### 3-(4,5-dimethylthiazol-2-yl)-2,5-diphenyltetrazolium bromide) tetrazolium (MTT) reduction assay on cell viability submitted to DETC

The ability of RAW 264.7 macrophages and fibroblast 3T3 to reduce MTT was evaluated according to the previously described method of Mosmann^53^. Initially, the cells were seeded in 96-well plates at a density of 5 × 103/well and kept at culture condition for 12 h. Then, the culture medium was replaced by another medium containing DETC in the different concentrations (from 4.444 to 2.222 µM). After 24 h, the culture medium was replaced with 100 μL of MTT (1 mg/mL dissolved in DMEM). Then, the cells were incubated for 4 h at 37 C. Subsequently, the culture supernatant was discarded, and the crystals of formazan were solubilized in ethanol, 100 μL/well. Absorbance was measured with an Epoch microplate spectrophotometer (Biotek Instruments Inc., Winooski, VT, USA) at 570 nm. Cell viability was calculated in relation to the negative control using the formula: % viability = (Atest/AControl) × 100, in which Atest corresponds to the absorbance of the experimental group, and Acontrol corresponds to the absorbance of the negative control. Based on % MTT reduction, IC_50_, that represents the drug concentration necessary to reduce by fifty per cent the cell population, was calculate. In order to analyse a possible chemical incompatibility between DETC and MTT, wells were analysed containing only the DMEM, DETC and MTT culture medium. It was observed that DETC does not interact chemically with MTT.

### Determination of selective index of DETC

To assess the predilection of DETC between the parasite and cell, the Selective Index (SI) was used. This Index represents the preference of drug calculated by: SI = IC_50_parasite/IC_50_cell; in which IC_50_ parasite represents the drug concentration to reduce by fifty per cent the parasite population in vitro and IC_50_ cell represents the drug concentration to reduce by fifty per cent the in vitro cell population.

### Evaluation of death mechanism in *Trypanosoma cruzi* caused by DETC

Annexin V/PI (propidium iodide) was used to evaluate the DETC death mechanism at *T. cruzi*. Briefly, the epimastigotes and trypomastigotes forms of the different strains of *T. cruzi*, in the same culture conditions described previously in topic 2.2, were exposed to DETC (4.44 µM, 44.40 µM or 444.00 µM) for 24 h. Then, the parasites were centrifuged (2000 rpm for 5 min, 4 C), rinsed with PBS 1 × pH 7.4 (Phosphate-Saline Buffer), and suspended in 300 µL bond buffer 1×. Then, 5 µL of Annexin V-FITC (fluorescein isothiocyanate) was added to the system, let reacting for 10 min. Then, the parasites were centrifuged (2000 rpm for 5 min, 4 C) and resuspended again in 200 µL Bind Buffer and 10 µL of PI. Actinomycin D (20.0 mM) was used as positive control. All these steps are described in the protocol proposed by the manufacturer (Annexin V-FITC Apoptosis Detection Kit, Invitrogen, Carlsbad, CA, USA). After 10 min, the fluorescence intensity was determined using a flow cytometer (FACSCanto II, BD Biosciences, Eugene, OR, USA) with FACSDiva software, version 6.1.2 (Becton Dickson, Franklin Lakes, NJ, USA). For the analysis, we counted 10,000 events for each strain and evolutionary form of *T. cruzi*. A solution of 100% DMSO was used as positive control using the same protocol of Annexin V/PI stain of DETC. The solution was added in the wells and the parasite was exposed for 24 h. Conditions were the same used in topic 2.2.

### Evaluation of mitochondrial damage in *Trypanosoma cruzi* by DETC

In order to analyse the mitochondrial damage induced in different strains and forms of *T. cruzi* by DETC, the mitochondrial potential marker, rhodamine-123 (Invitrogen, Carlsbad, CA, USA) was used, following the manufacturer's recommendations. Briefly, the different strains and forms of *T. cruzi* were treated with different concentrations of DETC (44.40 µM; 111.00 µM; or 222.00 µM) for 24 h in the same conditions described previously. After treatment, the parasites were centrifuged at 2000 rpm for 6 min at 4 C and rinsed with PBS pH 7.4. Then, the parasites were suspended and 200 µL of PBS and 0.5 µL of rhodamine-123 (5 mg/mL) were added, as described by Sulsen et al., 2016^54^. Carbonyl cyanide 3-chlorophenylhydrazone (CCCP; 100.0 μM) was used as positive control. After 30 min, the parasites were rinsed twice in PBS and analysed by flow cytometry at wavelengths of 488 and 633 nm. Finally, the fluorescence intensity was determined by flow cytometer (FACSCanto II, BD Biosciences, Eugene, OR, USA) with FACSDiva software, version 6.1.2 (Becton Dickson, Franklin Lakes, NJ, USA). The parasites mitochondrial activity was calculated according to the formula: reduction of probe attachment (%) = (fluorescence intensity of the parasites treated with DETC)/(fluorescence intensity of negative control) * 100. A total of 10,000 events were analysed for each strain and form of *T. cruzi*.

### Evaluation of reactive oxygen species production in *Trypanosoma cruzi* by DETC

In order to analyse the reactive oxygen species production in different strains of *T. cruzi* after treatment with DETC, the marker 2´,7´-dichlorofluorescin diacetate (Sigma, Saint Louis, MO, USA) was used^55^. Briefly, the different strains and forms of *T. cruzi* were treated with different concentrations of DETC (44.40 µM; 111.00 µM; or 222.00 µM) for 24 h in the same conditions described previously. After treatment, the parasites were centrifuged at 2000 rpm for 6 min at 4 C and washed with PBS pH 7.4. Afterward, these parasites were loaded with 10 µM of 2´,7´-dichlorofluorescin diacetate and maintained in dark for 45 min. The endogenous ROS hydrogen peroxide (H_2_O_2_) was used as a positive control 0.5 mM*.* The reactive oxygen species production was measured by increase of fluorescence caused by conversion of probe, and read at equipment GloMax^®^ Discover Microplate Read model GM 3000 (Fitchburg, Madison, USA) at λ_ex_ = 490–530.

### Statistical analysis of the data

All experiments were carried out in triplicate and independently. The results are presented in the form of arithmetic mean ± standard deviation. Data were submitted to the Shapiro–Wilk normality test. Parametric data were analysed using the ANOVA test associated with the Tukey-T post-test (for more of 2 groups), t-student test (for 2 groups), and Pearson correlation. Tests were performed using the software GraphPad Prism v. 7.0 (2016) and P.A.S.T v. 2.17 (2012) (https://palaeo-electronica.org/2001_1/past/issue1_01.htm).

## Supplementary Information

Supplementary Information.
