# Supplementary material for: Insights of antiparasitic activity of sodium diethyldithiocarbamate against different strains of Trypanosoma cruzi
Source: Sci Rep. 2021 May 27;11:11200. doi: 10.1038/s41598-021-90719-0 (PMC8159965; doi:10.1038/s41598-021-90719-0)
Supplement: Supplementary file 1 — Supplementary Information. [file 41598_2021_90719_MOESM1_ESM.pdf]

Insights of antiparasitic activity of sodium diethyldithiocarbamate against different strains of *Trypanosoma cruzi*

Johny Wysllas de Freitas Oliveira[1,2], Taffarel Melo Torres[3], Cláudia Jassica Gonçalves Moreno[1,2,4], Bruno Amorim-Carmo[2], Igor Zumba Damasceno[5], Ana Katarina Menezes Cruz Soares[6], Jefferson da Silva Barbosa[7], Hugo Alexandre Oliveira Rocha[2,6], and Marcelo Sousa Silva[1,2,4,8*]

[1]Immunoparasitology Laboratory, Department of Clinical and Toxicological Analysis, Centre of Health Sciences, Federal Universi-ty of Rio Grande do Norte, Natal, Brazil. johny3355@hotmail.com (J.W.F.O.);

[2]Programa de Pós-graduação em Bioquímica, Centro de Biociências, Universidade Federal do Rio Grande do Norte. Natal, Brazil. Bruno_portilo@hotmail.com (B.A.-C.);

[3]Centro de Ciências Biológicas e da Saúde- Universidade Federal Rural de Semi-árido. Mossoró, Brazil. taffa-rel.torres@ufersa.edu.br (T.M.T.);

[4]Programa de Pós-graduação em Ciências Farmacêuticas, Centro de Ciências da Saúde, Universidade Federal do Rio Grande do Norte. Natal, Brazil. claudia.mrn1@gmail.com (C.J.G.M.);

[5]Departamento de Engenharia de Materiais, Centro de Tecnologia, Universidade Federal do Rio Grande do Norte. Natal, Brazil. igorzumba@ufrn.edu.br (I.Z.D.);

[6]Laboratório de Biotecnologia de Polímeros Naturais – BIOPOL, Departamento de Bioquímica, Centro de Biociências, Universidade Federal do Rio Grande do Norte. Natal, Brazil. hugo@cb.ufrn.edu (H.A.O.R.); anakaty2018@gmail.com (A.K.M.C.S.);

[7]Instituto Federal de Educação, Ciência e Tecnologia do Rio Grande do Norte (IFRN) - Campus São Gonçalo do Amarante. São Gonçalo do Amarante, Brazil. jefferson.barbosa@ifrn.edu.br (J.S.B.);

[8]Global Health and Tropical Medicine, Instituto de Higiene e Medicina Tropical, Universidade Nova de Lisboa. Lisbon, Portugal. mssil@ihmt.unl.pt (M.S.S.).

Corresponding Author:

Prof. Marcelo Sousa Silva, Ph. D.

E-mail: mssilva@ihmt.unl.pt

Supplementary information:

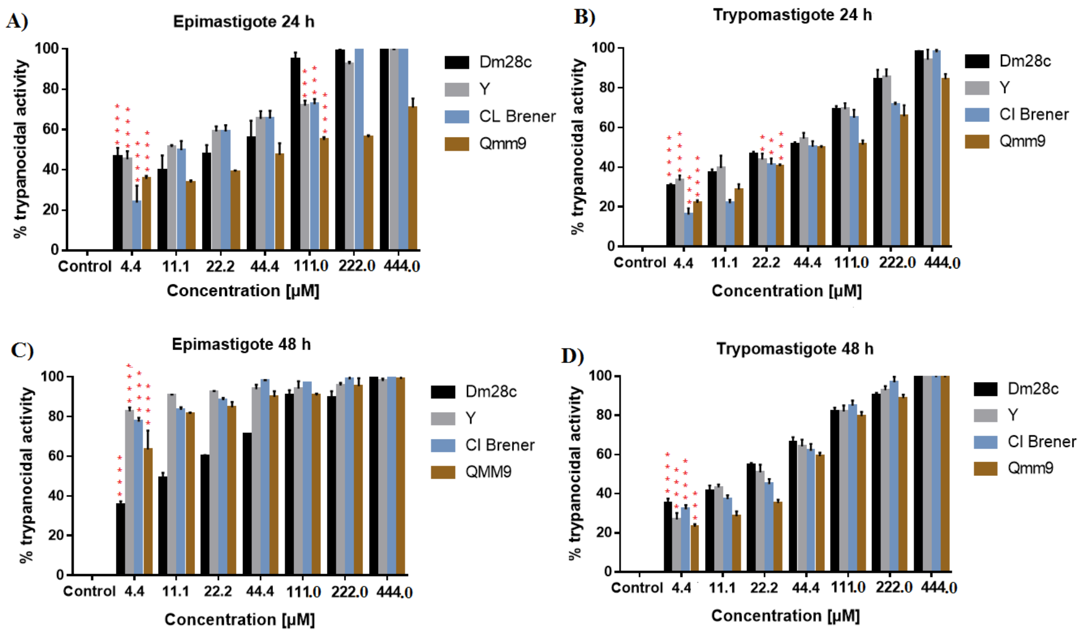

Figure 1S: Evaluation of resazurin reduction inhibition by different strains and forms of *Trypanosoma cruzi* treated with DETC. Parasitic inhibition of resazurin reduction (%) of DETC against different forms (epimastigotes and trypomastigotes) and strains (Y, Dm28c, CL Brener, and QMM9) of *T. cruzi*, after 24 and 48 hours of culture and analysed by resazurin assay. A: Epimastigotes for 24 hours of treatment; B: Trypomastigotes for 24 hours of treatment; C: Epimastigotes for 48 hours of treatment; D: Trypomastigotes for 48 hours of treatment. Results presented as mean ± standard deviation of the percentage of parasitic inhibition in a triplicate system and for the statistical analysis of the Anova Test, together with the Tukey Post-test (P <0.01 (**); P <0.001 (***), P<0.0001(****). In order to verify differences, the profile of each strain was compared against others treated with the same concentration of DETC. Software GraphPad Prism v. 7.0 (https://www.graphpad.com/) (2016).

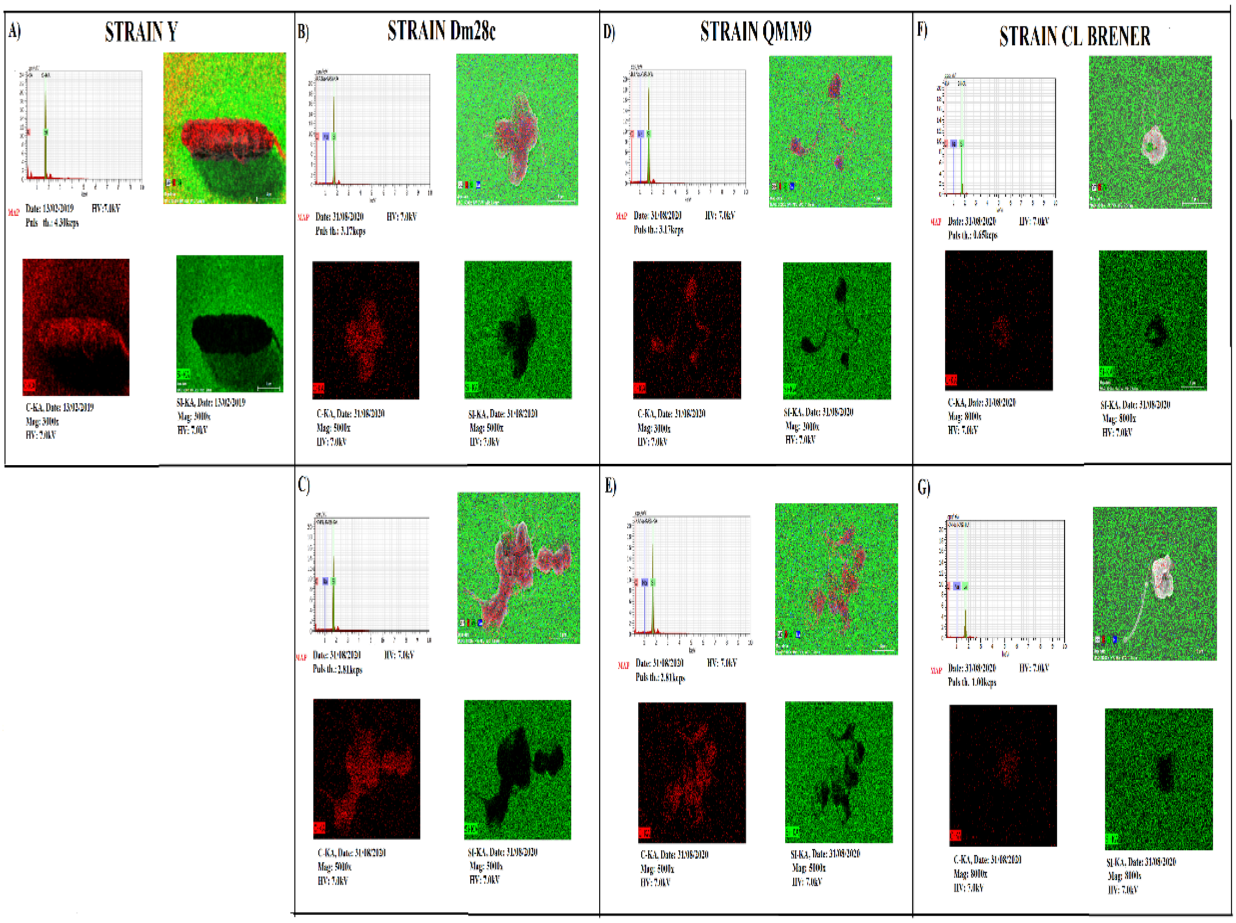

Figure 2S: Spectroscopy of energy dispersion (SED) of different MEV images after treatment with DETC to prove the mains chemical elements that compose the image. A) Image of parasite strain Y after treatment with DETC corresponding the images 3 in figure 2, respectively ; B) and C) images to refer strain Dm28c after treatment with DETC corresponding the images 7 and 8 in figure 2, respectively; D) and E) images to refer strain QMM9 after treatment with DETC corresponding the images 10 and 11 in figure 2, respectively; F) and G) images to refer strain CL Brener after treatment with DETC corresponding the images 16 and 15 in figure 2, respectively. Scanning electron microscopy under a FEG microscope (Model augira, Brand Carl Zeiss, Oberkochen, WB, GER).

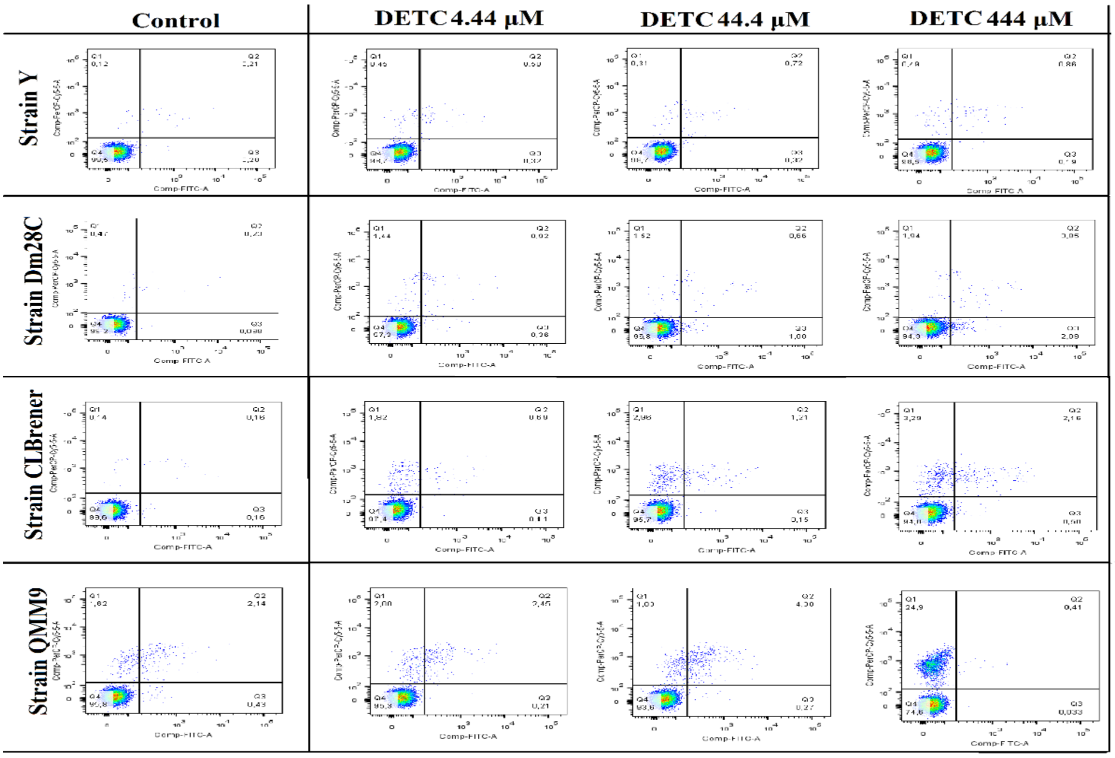

Figure 3S: Analysis of the cell death mechanism of epimastigote from different Trypanosoma cruzi strains treated with DETC. The parasites death cell mechanism was evaluated by flow cytometer as to the capacity for Annexin/Pi stain after 24 hours of treatment with different DETC concentrations; 4.44 µM; 44.44 µM; and 444.00 µM. Flow cytometer (FACSCanto II, BD Biosciences, Eugene, OR, USA) with FACSDiva software, version 6.1.2 (https://www.bdbiosciences.com/en-us/instruments/research-instruments/research-software/flow-cytometry-acquisition/facsdiva-software) (Becton Dickson, Franklin Lakes, NJ, USA).

Table 1S: DETC antiparasitic activity, expressed in IC_{50} values ± standard deviation, against the different strains and forms of *Trypanosoma cruzi* after 24 hours of exposure, based on results expressed by rezasurin metabolization essay. The statistical analysis the Anova Test together with the Tukey Post-test. The statistical analysis used measure the IC_{50} value in each group under same conditions.

***Trypanosoma cruzi* strains (DTUs)**	**IC_{50} epimastigote**		**IC_{50} trypomastigote**	
	**DETC (µM)**	**BZN (µM)**	**DETC (µM)**	**BZN (µM)**
Strain Dm28c **(TcI)**	15.94±7.43 [b]	69.32±8.42 [a]	25.00±5.34 [a]	89.95±4.87 [a]
Strain Y **(TcII)**	9.44±3.181 [a]	92.66±7.78 [b]	23.35±5.41 [a]	98.74±3.98 [b]
Strain QMM9 **(TcIII)**	60.49±7.62 [c]	99.38±9.41 [b]	53.49±8.47 [b]	108.71±6.74 [c]
Strain CL Brener **(TcVI)**	15.18±3.64 [b]	62.86±8.47 [a]	43.18±7.61 [b]	82.43±5.83 [a]

BZN - benznidazole
